# Spatio-Temporal Joint Trajectory Planning for Autonomous Vehicles Based on Improved Constrained Iterative LQR

**DOI:** 10.3390/s25020512

**Published:** 2025-01-17

**Authors:** Qin Li, Hongwen He, Manjiang Hu, Yong Wang

**Affiliations:** 1 National Engineering Laboratory for Electric Vehicles, Beijing Institute of Technology, Beijing 100081, China; qinlee1993@163.com (Q.L.); 17862709675@163.com (Y.W.); 2 State Key Laboratory of Advanced Design and Manufacturing Technology for Vehicle, Changsha 410082, China; manjiang_h@vip.163.com

**Keywords:** trajectory planning, constrained iterative LQR, autonomous driving

## Abstract

With advancements in autonomous driving technology, the coupling of spatial paths and temporal speeds in complex scenarios becomes increasingly significant. Traditional sequential decoupling methods for trajectory planning are no longer sufficient, emphasizing the need for spatio-temporal joint trajectory planning. The Constrained Iterative LQR (CILQR), based on the Iterative LQR (ILQR) method, shows obvious potential but faces challenges in computational efficiency and scenario adaptability. This paper introduces three key improvements: a segmented barrier function truncation strategy with dynamic relaxation factors to enhance stability, an adaptive weight parameter adjustment method for acceleration and curvature planning, and the integration of the hybrid A* algorithm to optimize the initial reference trajectory and improve iterative efficiency. The improved CILQR method is validated through simulations and real-vehicle tests, demonstrating substantial improvements in human-like driving performance, traffic efficiency improvement, and real-time performance while maintaining comfortable driving. The experiment’s results demonstrate a significant increase in human-like driving indicators by 16.35% and a 12.65% average increase in traffic efficiency, reducing computation time by 39.29%.

## 1. Introduction

In recent years, the continuous development of autonomous driving technology has led to the application of autonomous driving functions in an increasing number of complex scenarios [1]. Trajectory planning is one of the critical technologies in autonomous driving [2]. By receiving upstream information, including obstacles, maps, decision results, and localization information, trajectory planning outputs a safe, smooth, and comfortable driving path point sequence, which maintains real-time performance. The trajectory directly impacts motion control output, thereby influencing the vehicle’s lateral and longitudinal dynamic movements [3]. Therefore, trajectory planning has a crucial impact on the comfort, safety, and human-like behavior of autonomous driving [4].

However, achieving autonomous driving in complex urban environments still presents several challenges. First, urban driving conditions are complex and variable, requiring trajectory planning that can effectively manage complex nonlinear constraints and respond in real time to dynamic obstacles [5]. Second, simultaneous optimization of temporal and spatial constraints is critical to meet the planned trajectory that meets safety, comfort, and human-like driving performance [6]. Additionally, it is crucial to ensure computational efficiency to enhance real-time performance.

To address challenges mentioned above, many scholars have conducted research on trajectory planning methods, including sampling-based, interpolation, and graph search techniques. While the problem of interpolation and sampling-based trajectory planning methods ensures solving efficiency, complex constraints are hardly involved in the problem-solving process [7]. In complex urban environments with multiple dynamic obstacles, sampling-based methods incur significant resource overhead [8]. The graph search method is flexible and highly generalizable; higher grid resolutions enhance the dynamic performance of autonomous vehicles [9], but they can severely impact real-time capabilities, especially in complex scenarios with multiple dynamic obstacles. Each execution cycle updates the grid map to account for dynamic obstacles, leading to significant computational overhead. The heuristic method [10] offers high adaptability and flexibility in complex environments and can achieve global optimization. However, its lack of model constraints and poor real-time performance, as seen in methods like GBSO, B-spline curves fail to satisfy vehicle model constraints under complex urban conditions [11].

Advancements in vehicle computing capabilities and machine learning now enable real-time deployment of neural network models on vehicle computing platforms to generate trajectories [12]. Neural networks have been successfully applied in path planning and provide a strong foundation for autonomous vehicle path planning [13,14]. Scholars have trained trajectory planning models using techniques such as imitation learning [15], reinforcement learning [16], deep reinforcement learning [17], and inverse reinforcement learning [18], validating them in simulations and real-world vehicles [19]. Neural networks inherently excel at addressing complex nonlinear issues, although validation results have been successful in specific scenarios; driving safety still cannot be assured, and interpretability issues persist.

Numerical optimization is the most widely used approach for trajectory planning, effectively addressing the aforementioned challenges. It formulates the planning problem as an optimization problem for constrained vehicle nonlinear dynamic systems, generating trajectories that meet constraints through dynamic mathematical optimization. This approach primarily includes nonlinear programming, convex optimization, and optimal control. Solution methods can be classified into two categories: direct and indirect approaches. Direct methods treat current trajectory points as decision variables, optimizing the trajectory by directly modifying these points. A common example is Sequential Quadratic Programming (SQP), typically implemented within a model predictive control framework that integrates various constraints and problem formulations for trajectory planning.

It is important to clarify that, in terms of constraint handling, trajectory planning can be classified into two types: spatio-temporal joint planning and spatio-temporal decoupled planning, or denoted as path velocity decomposition (PVD) and non-PVD method. The former converts a 3D problem into two 2D ones, which largely facilitates the solution process, and the latter directly plans trajectories, which has the underlying merit to improve the solution optimality, taking into account both lateral and longitudinal spatio-temporal constraints. This approach allows for a more comprehensive optimization of the vehicle’s trajectory, ensuring that it meets various constraints in both space and time [20].

Lin et al. [5] developed a trajectory planning strategy for emergency situations by integrating a model predictive controller with potential functions. This approach incorporates a sigmoid-based safety corridor into the MPC constraints to plan trajectories for emergency obstacle avoidance scenarios. Long Short-Term Memory (LSTM) networks, a type of deep neural network (DNN), are capable of extracting sequential features and managing complex dependencies. When combined with model predictive control, they improve prediction accuracy and are integrated into deep deterministic policy gradient algorithms, enhancing the robustness and efficiency of path planning, speeding up convergence, and reducing failure rates [21]. Several researchers have combined model predictive control with reinforcement learning methods. Additionally, deep reinforcement learning (DRL) components provide the capability to learn from experience and optimize control performance over time, allowing adaptation to new situations and real-time performance enhancement [22,23,24]. Zuo et al. [25] proposed a progressive model predictive control framework for trajectory planning, integrating model predictive control with artificial potential fields (APFs). This approach treats time-varying safety constraints as repulsive forces and uses an asymmetric lane potential field function to generate obstacle avoidance paths. Velocity planning is subsequently conducted along the path, and the resulting trajectories are utilized for trajectory tracking control. Svensson et al. [26] introduced an adaptive MPC-based trajectory planning method for situations where obstacles suddenly appear in front of the vehicle. This method updates the constraints in the optimization function based on real-time vehicle data, resulting in trajectories that consider time-varying tire friction constraints. However, a significant drawback of MPC-based trajectory planning is its high computational complexity. Each optimization cycle necessitates calculating the optimal trajectory over the entire prediction horizon, resulting in considerable resource waste, and it fails to ensure real-time performance for spatiotemporal trajectory planning issues.

Indirect methods generally use control inputs as decision variables, deriving the optimal trajectory through the forward propagation of the system’s dynamic equations. Differential dynamic programming (DDP) effectively addresses unconstrained optimal control problems [27] and manages upper and lower bounds on control inputs. Derived from dynamic programming theory and linear quadratic regulators (LQRs), iterative linear quadratic regulators (ILQRs) are well-suited for optimizing nonlinear systems and have demonstrated enhanced computational efficiency [28]. Chen et al. [29] introduced a CILQR by integrating comfort and safety constraints into the ILQR optimization problem. This approach enables motion planning for autonomous vehicles by improving the obstacle function to incorporate hard constraints into the optimization process [30]. Pan et al. [31] proposed a two-stage uncertainty-aware prediction method to improve the adaptability of trajectory planning across various scenarios. Building on a single-track model, Li et al. [32] improved the reliability of the kinematic model and ensured trajectory executability by incorporating higher-order terms, curvature rates, and longitudinal impacts. CILQR demonstrates advantages in tackling spatio-temporal trajectory planning problems, efficiently handling nonlinear complex constraints while maintaining high solving efficiency and rapid iteration speeds.

Nevertheless, certain aspects still require further improvement. To address these issues, we propose an improved CILQR method with three key features. (1) A new barrier function is introduced to mitigate stability challenges caused by excessive gradient variations in traditional barrier functions. (2) An adaptive weight parameter adjustment strategy is developed, enabling dynamic adjustment of the cost function weights based on vehicle states, thereby improving overall dynamic performance. (3) The hybrid A* algorithm is leveraged to create a new initial trajectory optimization approach, which generates collision-free initial trajectories more quickly, enhancing trajectory planning efficiency.

These improvements make the CILQR method more applicable to real-world autonomous driving scenarios. The proposed approach is validated through simulations and real-vehicle experiments across various scenarios, including the continuous nudge static obstacle, dynamic cut-in obstacle collision avoidance, dynamic overtaking of oncoming obstacles, and collision avoidance with dynamic and static obstacles. The results demonstrate that the enhanced CILQR effectively addresses complex driving situations that the original method struggles with. It is shown to be well-suited for autonomous driving in challenging urban environments, ensuring safety, comfort, and human-like driving behavior. The rest of this paper is organized as follows: Section 2 describes the problem, detailed features, the framework proposed in this paper, and trajectory optimization and constraints. Simulations, real-vehicle experiments, and analysis results are introduced in Section 3. The article concludes in Section 4.

## 2. Proposed Method

### 2.1. ILQR Method

Assume the vehicle model is a nonlinear discrete system described by $x_{k+1}=f(x_{k},u_{k})$, where $x_{k},u_{k}$ denote the state and control sequences at time k, respectively. To tackle the trajectory planning problem, the ILQR method can be utilized for optimization based on the initial trajectory $\left(x_{k},u_{k}\right)$ provided from upstream. The goal is to identify a sequence of states and control inputs that minimizes the cost function.

(1) $$\begin{array}{c} \underset{\begin{array}{c} u_{0},\ldots ,u_{N-1}\\ x_{0},\ldots ,x_{N} \end{array}}{min}J=L(x_{N})+\sum_{k=0}^{N-1}L\left(x_{k},u_{k}\right)\\ s.t.\;\;x_{k+1}=f(x_{k},u_{k}),\;x_{0}=x_{start} \end{array}$$

where N denotes the number of optimization iterations, $L(x_{N})$ is the cost function at time N, and $x_{start}$ refers to the initial planning point.

ILQR is an algorithm used to solve optimal control problems for nonlinear systems [33]. At each time step, it linearizes the system’s state equations using a Taylor expansion and approximates the cost function with a second-order expansion. The LQR is then applied to solve this approximated problem. By iteratively adjusting the control policy, the algorithm gradually converges to the optimal strategy. The solution to the ILQR problem primarily involves two components: the backward pass and the forward pass. The backward pass focuses on optimizing the control increments, while the forward pass updates the current trajectory based on the control increments and the vehicle model. We will first derive the computation process for the backward pass function. According to Bellman’s principle, we can define:

(2) $$V_{N}(x_{N})\&=L(x_{N})$$

(3) $$V_{k}(x_{k})\&=\underset{u}{min}\{L(x_{k},u_{k})+V_{k+1}(f(x_{k},u_{k}))\}$$

where $V_{k}(x_{k})$ is the optimal cost-to-go start at k step, considering a small Perturbation, and define $Q(x_{k},u_{k})=L(x_{k},u_{k})+V_{k+1}(f(x_{k},u_{k}))$, $V_{k}(x_{k})=\underset{u}{min}\{Q(x_{k},u_{k})\}$, by ignoring higher-order terms, getting an approximation of the Perturbation. The approximation of the Perturbation function $\delta Q\left(x_{k},u_{k}\right)$ is convex with respect to the action increment as follows:

(4) $$\nabla_{\delta u_{k}}\delta Q\left(x_{k},u_{k}\right)=0$$

We have the optimal action increment with respect to the optimal state increment:

(5) $$\left\{\begin{array}{c} \delta u^{*}=-Q_{uu}^{-1}\left(Q_{ux}\delta x_{k}+Qu\right)=K\delta x+d\;\\ d=-Q_{uu}^{-1}Q_{u}\\ K=-Q_{uu}^{-1}Q_{ux}\; \end{array}\right.$$

We can represent the optimal k-th step Perturbation with respect to δx and get V first-order, second-order term, and increment:

(6) $$\left\{\begin{array}{c} \frac{\partial V}{\partial x}=Q_{x}+K^{T}Q_{uu}d+K^{T}Q_{u}+Q_{ux}^{T}d\\ \frac{\partial^{2}V}{\partial x^{2}}=Q_{xx}+K^{T}Q_{uu}K+K^{T}Q_{ux}+Q_{ux}^{T}K\\ \Delta V=\frac{1}{2}d^{T}Q_{uu}d+d^{T}Q_{u} \end{array}\right.$$

At this stage, a single iteration of the backward pass is completed, and the iterative loop will persist until the cost function triggering condition is satisfied or until N=1. Each backward pass determines the optimal feedback gain for each time step, updating the state and control sequences ${\bar{x}}_{k},{\bar{u}}_{k}$, according to the vehicle model. This process is referred to as the forward pass and is derived as follows:

(7) $$\left\{\begin{array}{c} x_{0}=x_{start}\\ {\bar{u}}_{k}=u_{k}+\delta u_{k}\\ {\bar{x}}_{k+1}=f\left({\bar{x}}_{k},{\bar{u}}_{k}\right)\; \end{array}\right.$$

where ${\bar{x}}_{k},{\bar{u}}_{k}$ denote the updated trajectory and control sequences, respectively. Due to approximations made in calculating the feedback gains, a line search method can further minimize the objective function. Typically, a backtracking approach is employed, referring to Equation (5), resulting in $\delta u_{k}=K\delta x+\alpha d$. Throughout the iterations, α is continually adjusted by multiplying it by a fixed ratio, α=ρα, where 0<ρ<1, thereby yielding a more optimal solution and avoiding overshoot.

### 2.2. Improved CILQR Method

**Figure 1:**
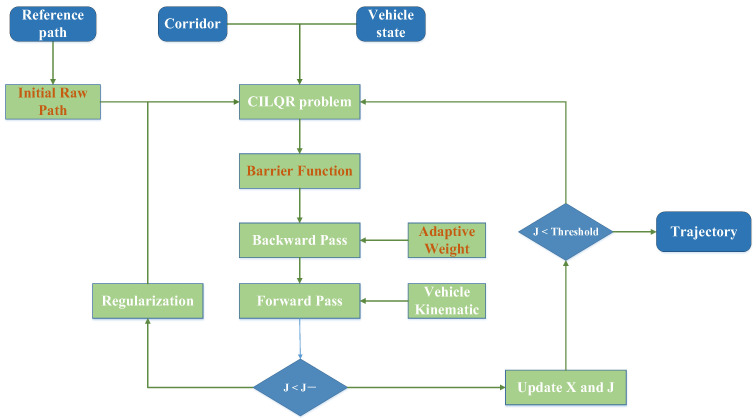
Improved CILQR process.

ILQR depends on linearizing the system dynamics and applying a quadratic approximation to the cost function. This approach is effective for problems with little to no nonlinearity or constraints. However, when faced with complex nonlinearity and strict constraints, these approximations can result in greater linearization errors or inaccuracies in the quadratic approximation, leading to non-convergence of the optimization problem [34]. ILQR struggles with direct constraint handling since it lacks an inherent mechanism for this purpose. Moreover, added constraints apply only to specific time steps, and cumulative changes in trajectory during the forward pass can lead to constraint violations, complicating the management of complex control and state constraints. CILQR transforms inequality constraints into penalty terms and integrates them into the cost function, enabling the use of the ILQR algorithm framework for solving complex constraint problems, as shown in Figure 1. The improved CILQR process with three key features, which are marked in red, is presented. The CILQR problem can be expressed as follows:

(8) $$\begin{array}{c} \underset{\begin{array}{c} u_{0},\ldots ,u_{N-1}\\ x_{0},\ldots ,x_{N} \end{array}}{min}J=L(x_{N})+\sum_{k=0}^{N-1}L\left(x_{k},u_{k}\right)\\ s.t.x_{k+1}=f\left(x_{k},u_{k}\right),x_{0}=x_{start}\\ g_{k}(x_{k},u_{k})<0,g_{N}(x_{N})<0 \end{array}$$

where $g_{k}\left(x_{k},u_{k}\right)$ denotes the inequality constraints on the state and control variables at time k, while $g_{N}(x_{N})$ indicates the terminal state constraint.

An ideal barrier function would be an indicator function, yielding a cost of zero when $g_{k}(x_{k},u_{k})<0$ and infinite otherwise. However, its non-differentiability prevents the calculation of the Hessian matrix. Consequently, it is commonly approximated using logarithmic or exponential functions, with modulation parameters ensuring it approaches positive infinity within a designated calibrated range, thereby achieving a hard constraint representation, which can be theoretically validated for convergence. For example, the typical forms of barrier functions utilizing exponential and logarithmic functions are as follows:

(9) $$b_{k\_1}^{x}=q_{1}exp(q_{2}g_{k}(x_{k},u_{k}))$$

(10) $$b_{k\_2}^{x}=q_{3}{log}_{q_{4}}(q_{5}g_{k}(x_{k},u_{k}))$$

where $q_{1}\sim q_{5}$ denote the parameters used to modulate the barrier function.

Regardless of the chosen barrier function, stability issues arise, leading to a trade-off between rigid obstacle avoidance constraints and numerical stability. If the barrier function’s curve changes too gently, it can result in violations of the constraints, breaching the hard constraints. Conversely, if the curve changes too abruptly, the barrier function may tend toward infinity near the constraint boundaries, leading to extreme sensitivity in the optimization problem and causing numerical solutions to become unstable or difficult to converge. While this characteristic of the barrier function is beneficial for ensuring obstacle avoidance safety, it simultaneously raises computational complexity and instability.

To resolve these challenges, a truncation strategy for the barrier function is established to avoid drastic gradient changes near the boundaries that could impact system stability. Given that the barrier function is generally monotonically increasing, upper and lower limits are enforced on its derivative. Furthermore, a piecewise strategy is employed to improve adaptability to various scenarios, and a dynamic relaxation factor is introduced to enhance stability in the optimization process.

(11) $$\left\{\begin{array}{c} b_{k\_3}^{x}=q_{max},\;\nabla b_{k\_2}^{x}>q_{up}\\ b_{k\_3}^{x}=b_{k\_2}^{x}+\epsilon_{k}\left(x_{k},u_{k}\right),q_{low}<\nabla b_{k\_2}^{x}\leq q_{up}\\ b_{k\_3}^{x}=q_{min},\nabla b_{k\_2}^{x}\leq q_{low} \end{array}\right.$$

where $q_{min},q_{max}$ denote the lower and upper bounds of the barrier function values, respectively, $q_{low}$, $q_{up}$ denote the lower and upper limits of the reciprocal of $b_{k\_2}^{x}$, respectively, and $\epsilon_{k}\left(x_{k},u_{k}\right)$ is the function of state and control sequence. To ensure second differentiability at the segmentation points, cubic spline interpolation is commonly used. This facilitates a second-order Taylor expansion, which is subsequently utilized during the backward pass for optimization.

The expected behavior of the vehicle in interaction with obstacles varies with factors such as current speed, relative speed, distance, road curvature, and obstacle type. For instance, at higher speeds, to maintain vehicle stability, the vehicle will strive to avoid generating significant lateral acceleration while allowing for greater control margins and planning a smoother curvature change in the trajectory. Consequently, it is necessary to dynamically adjust the weight parameters for lateral acceleration and curvature based on the current planned speed during the iterative process.

(12) $$w_{ax}=p_{ax_{1}}\frac{v_{x_{n}}}{exp[p_{ax_{2}}\left(a_{x_{n}}^{2}+v_{x_{n}}^{4}\kappa_{x_{n}}^{2}\right)]}+p_{ax_{3}}$$

(13) $$w_{\dot{\kappa }}=p_{{\dot{\kappa }}_{1}}\frac{v_{x_{n}}}{exp[p_{{\dot{\kappa }}_{2}}a_{x_{n}}^{2}]}+p_{{\dot{\kappa }}_{3}}\frac{∆v_{n}}{exp[p_{{\dot{\kappa }}_{4}}∆x_{n}]}+p_{{\dot{\kappa }}_{5}}$$

where $p_{ax_{1}}\sim p_{ax_{3}}$ and $p_{{\dot{\kappa }}_{1}}\sim p_{{\dot{\kappa }}_{5}}$ are calibratable parameters, and $v_{x_{n}},a_{x_{n}},\kappa_{x_{n}},∆v_{n}$, and $∆x_{n}$ denote the vehicle speed, longitudinal acceleration, curvature, velocity difference with the target vehicle, and relative distance at the n-th trajectory point, respectively. Current dynamic control margins are represented by lateral and longitudinal accelerations. Based on the kinematic assumption $v_{y_{n}}=v_{x_{n}}^{2}\kappa_{x_{n}}$, when the acceleration vector of the planned trajectory is large, a penalty term for longitudinal acceleration requests is increased, which also grows with the current speed. Conversely, when the longitudinal acceleration is high and relative speed and distance are low, the penalty term for curvature change rate is also increased, scaling with the current speed.

The quality of the initial trajectory significantly impacts the reduction in iteration count, enhances solution efficiency, and ensures the real-time performance of trajectory planning. The initial trajectory is generated using the hybrid A* method, followed by speed planning based on simple kinematic equations and interpolation techniques. The hybrid A* algorithm combines Dijkstra’s algorithm and breadth-first search (BFS). The heuristic function h(n) needs to account for both the cost from the current point to the target point and the cost associated with deviations in heading angle to enable initial obstacle avoidance. Therefore, it can be defined as follows:

(14) $$h(n)={\left((x-x_{goal}{)}^{2}+(y-y_{goal}{)}^{2}+p_{h}(\theta -\theta_{goal}{)}^{2}\right)}^{\frac{1}{2}}$$

where $p_{h}$ represents the weight for heading angle errors, which is increased when lateral avoidance of obstacles is required or when approaching the target point. If the curvature of the reference trajectory exceeds a certain threshold, indicating that the vehicle must execute turns or U-turns, the cost related to heading angle errors can be ignored. Consequently, $p_{h}$ can be expressed as follows:

(15) $$p_{h}=\left\{\begin{array}{l} p_{h_{s}}\;\;\;\;\;,\mathrm{if}\;\lambda <\lambda_{l}\\ \frac{p_{h_{s}}(\lambda_{u}-\lambda )}{\lambda_{u}-\lambda_{l}},\;\;\mathrm{if}\;\lambda_{l}<\lambda <\lambda_{u}\\ 0,\;\;\;\;\;\;\;\mathrm{if}\;\kappa >\kappa_{c} \end{array}\right.$$

where λ represents the ratio of the distance error to the obstacle and the angle error at the current node and $\lambda_{l},\;\lambda_{u}$ denote the lower and upper limits, respectively. $\kappa_{c}$ denotes the upper limit of curvature for turning scenarios.

To enhance the efficiency of Hybrid A* searches in driving scenarios and reduce invalid searches caused by excessive heading angles, heading angle constraints are applied by considering the kinematic characteristics of the vehicle and road edges. Special attention is given to obstacle positions when constraining branching directions of the heading angle.

(16) $$\left|\Delta \theta |\leq min\;(abs\left(\theta_{R}\right),\tan^{-1}\left(\frac{L}{R_{min}}\right)\right)$$

where $\theta_{R}$ denote the heading angle from the ego vehicle to the road edge, the second term on the right side of the equation denotes the limitation of the vehicle kinematic, and $R_{min}$ denote the minimum of the turning radius.

The improved CILQR algorithm structure has been developed by refining the barrier function, adaptive weight adjustment methods, and initial trajectory optimization. First, the current raw trajectory is optimized by using the hybrid A* method to generate a basic obstacle avoidance path, thus enhancing the efficiency of trajectory iteration. After initializing the trajectory, a warm start is applied to initialize the matrix, which reduces stability issues during the backward pass. Prior to conducting the backward pass solution, the inequality constraints must be converted into cost function terms. During each loop of the backward pass iteration, the weight coefficients will be updated to match the cost function calculations at each path point. The loop termination conditions mainly take into account several factors, such as the cost function difference between iterations, the set maximum iteration count, and the rate of change in control gains. The pseudocode for the CILQR algorithm is presented in Algorithm 1.
**Algorithm 1.** Improved CILQR Algorithm1**:** **Output**: Trajectory vector and control sequence x,u2**:** //enable hybrid A* method; using the PI controller, get the speed profile3**:** **InitRefPath**: $x_{0},u_{0}$, tolerance4**:** // calculate the matrix in advance based on the initial trajectory to prevent oscillations5**:** **Warmstart**: $\delta Q\left(x_{0},u_{0}\right)$ ← $x_{0},u_{0}$6**:** **While** | ΔJ | < tolerance || stepcounter > threshold || feedforward gain ≈ 07**:**   **do**8**:**   $b_{k\_3}^{x},b_{k\_3}^{u}$ ← $g_{k}(x_{k},u_{k})$ // convert inequality constraints into cost functions9**:**   $J^{-}$ ← J
10**:**    K,d,ΔV ← **BACKWARDPASS**(x,u) 11**:**     AdaptiveWeight: $w_{ax},w_{\dot{\kappa }}$ // adaptive weight tuning function12**:**    X,U,J ← **FORWARDPASS**($x,u,K,d,\Delta V,J^{-}$)13**:**     α=ρα14**:** **return** x,u,J

### 2.3. Problem Formulation

The trajectory planning problem is established using a kinematic model. To account for lateral and longitudinal coupling simultaneously, the vehicle model is defined as $X_{k+1}=f\left(X,U\right)$, where $X={\left[x,y,v_{x},a_{x},\psi ,\kappa \right]}^{T}$ denotes the state vector and $U={\left[j_{x},\dot{\kappa }\right]}^{T}$ denotes the control vector [35]. The updated states are given as follows:

(17) $$\begin{array}{c} x_{k+1}=x_{k}+cos\left(\psi_{k}\right)ds\\ y_{k+1}=y_{k}+sin\left(\psi_{k}\right)ds\\ v_{x}^{k+1}=v_{x}^{k}+a_{x}^{k}T+\frac{1}{2}j_{x}^{k}T^{2}\\ a_{x}^{k+1}=a_{x}^{k}+j_{x}^{k}T\\ \psi_{k+1}=\psi_{k}+\kappa ds+\frac{1}{2}\left(\kappa_{k}a_{x}^{k}+v_{x}^{k}\dot{\kappa }\right)T^{2}+\frac{1}{3}\left(\frac{1}{2}\kappa_{k}j_{k}+a_{x}^{k}\dot{\kappa }\right)T^{3}+\frac{1}{8}\dot{\kappa }jT^{4}\\ \kappa_{k+1}=\kappa_{k}+\dot{\kappa }T \end{array}$$

where T denotes the time step. Assuming that the longitudinal jerk $j_{x}$ and the curvature rate $\dot{k}$˙ vary linearly and remain constant during time T, we have $j_{x}=da_{x}/dT$ and $\dot{k}=dk/dT$. By applying the small-angle assumption for the vehicle’s movement distance ds, we can obtain:

(18) $$ds=v_{x}T+\frac{1}{2}a_{x}T^{2}+\frac{1}{6}j_{x}T^{3}$$

To simplify the model, some researchers assume constant curvature k, resulting in $\psi_{k+1}=\psi_{k}+kds$. However, if the curvature rate is not considered, abrupt curvature changes can occur during forward propagation, which negatively impacts comfort and safety. We then differentiate the lateral jerk of the vehicle:

(19) $$j_{y}=\frac{d}{dt}\left(v_{x}^{2}\kappa \right)=2v_{x}\kappa a_{x}+\frac{v^{2}}{L}{sec}^{2}\left(\delta \right)\dot{\delta }=2v_{x}\kappa a_{x}+v_{x}^{2}\dot{\kappa }$$

The above equation suggests that the curvature rate can be approximated as the steering wheel rate, which justifies setting $\dot{\kappa }$ as a control input. To guarantee the feasibility of the planned trajectory, steering speed limits vary slightly with different velocities, and the vehicle’s minimum turning radius constraint must also be accounted for. Thus, the steering angle input can be substituted with a curvature rate constraint as the lateral control input, ensuring both controllability and comfort.

(1) Cost function-distance to the reference line

The reference line serves as a rough solution generated from decision-making processes informed by environmental perception. Excessive deviation from this line may pose safety risks. At time step k, the objective function is formulated as follows:

(20) $$c_{r}(k)=w_{r}{\left({\left(x-x_{r}\right)}^{2}-{\left(y-y_{r}\right)}^{2}\right)}^{2}$$

where $x_{r},y_{r}$ denote the coordinates of the closest point on the reference line derived from upstream inputs and $w_{r}$ is a calibratable weight. x,y denote the longitudinal and lateral coordinates at time step k, To enhance clarity, subscripts k have been omitted from all state and control variables in the objective function.

(2) Cost function-lateral motion

The lateral movement of the vehicle plays a crucial role in its comfort and safety. Consequently, the objective function is designed to consider the vehicle’s lateral speed, lateral acceleration, and lateral jerk:

(21) $$\begin{array}{c} c_{vy}\left(k\right)=w_{vy}{\left(v_{x}\left(\psi -\psi_{ref}\right)\right)}^{2}\\ c_{ay}\left(k\right)=w_{ay}{\left(v_{x}^{2}\kappa \right)}^{2}\\ c_{jy}(k)=w_{jy}{\left(2a_{x}v_{x}\kappa +v^{2}\dot{\kappa }\right)}^{2} \end{array}$$

where $\psi_{ref}$ denotes reference heading angle calculated from the reference line. Assuming the heading angle error is minimal, we approximate $v\left(\psi -\psi_{ref}\right)$ as the vehicle’s lateral speed. The parameters $w_{vy},w_{ay},w_{jy}$ are calibratable weights.

(3) Cost function-longitudinal motion

To ensure the vehicle’s longitudinal comfort and safety, it is crucial to consider the objective function related to the vehicle’s longitudinal motion, focusing on longitudinal speed, acceleration, and jerk:

(22) $$\begin{array}{c} c_{vx}\left(k\right)=w_{vx}{\left(v_{x}-v_{ref}\right)}^{2}\\ c_{ax}\left(k\right)=w_{ax}a_{x}^{2}\\ c_{jx}(k)=w_{jx}j_{x}^{2} \end{array}$$

where $v_{ref}$ denotes the reference speed initially assigned using a heuristic algorithm, which takes into account the set speed, the current vehicle speed, and the speeds of surrounding dynamic obstacles. The parameters $w_{vy},w_{ay},w_{jy}$ are calibratable weights.

(4) Cost function-trajectory curvature

The steering wheel rate can be approximated as the curvature rate. Rapid steering can significantly reduce comfort. Thus, we define the objective function for the curvature rate as follows:

(23) $$c_{\kappa }(k)=w_{\dot{\kappa }}{\dot{\kappa }}_{x}^{2}$$

where $w_{\dot{\kappa }}$ denotes the calibratable weight of curvature.

The inequality constraints primarily encompass collision risk constraints for safety, specifically the distance between the bounding boxes of static and dynamic obstacles and the inflated body of the vehicle:

(24) $$\sum_{i=0}^{N}D_{safe}-\left(P_{i}-P_{i}^{matched}\right)W_{C\_1}\left(P_{i}-P_{i}^{matched}\right)\geq D_{C\_1}$$

where $D_{safe}$ considers the safety distance between the vehicle and obstacles at various speeds. The terms $P_{i},P_{i}^{matched}$ represent the distances between the vehicle’s current location and the nearest matched point, typically corresponding to the front and rear safety positions. $W_{C\_1}$ is a calibratable matrix.

Given the capability limits of the vehicle’s chassis actuators—such as maximum turning radius, steering wheel speed, and drive torque—it is possible to derive corresponding state constraint boundaries based on the system’s defined limits. Furthermore, by considering the vehicle’s stability boundaries, constraints on maximum lateral acceleration and yaw rate can be established; the following constraints must be satisfied:

(25) $$\left\{\begin{array}{c} \begin{array}{c} v_{x}<v_{max}\\ a_{min}<a_{x}<a_{max}\\ j_{min}<j_{x}<j_{max} \end{array}\\ \begin{array}{c} \left|\psi -P(\psi )\right|<\psi_{diff\_max}\\ \left|\dot{\kappa }\right|<{\dot{\kappa }}_{max}\\ \left|\kappa \right|<\kappa_{max} \end{array}\\ \begin{array}{c} \left|\kappa v_{x}^{2}\right|<a_{y\_max}\\ \left|2\kappa a_{x}v_{x}+\dot{\kappa }v_{x}^{2}\right|<j_{y\_max}\\ \left|\kappa \cdot a+v\cdot \dot{\kappa }\right|<\omega_{max} \end{array} \end{array}\right.$$

where P(ψ) denotes the trajectory heading angle at the current matched point. It is essential to address the heading angle error to enhance continuity and possibility in scenarios with small turning radii. Under the assumptions of the vehicle’s kinematic model, $\kappa v_{x}^{2}$ can be approximated as lateral acceleration, while $2\kappa a_{x}v_{x}+\dot{\kappa }v_{x}^{2}$ can be similarly equated to lateral jerk. Considering $\ddot{\psi }\approx \kappa \cdot a+v\cdot \dot{\kappa }$, constraints can be applied to the vehicle’s yaw rate.

## 3. Experiment Validation

The efficacy of the improved CILQR method is demonstrated through the establishment of a simulation and real-vehicle testing platform. This paper details the development of an autonomous driving system based on ROS, deployed on a high-performance workstation equipped with an Intel Core i9-10900K processor (Intel, Shanghai, China), running a suitable Linux system to facilitate both simulation and real-vehicle tests.

During the simulation tests, data from real vehicles is collected to construct simulation scenarios in OSM (OpenStreetMap) format. Using actual vehicle parameters and the layered decoupling principle, a high-fidelity vehicle dynamics model is created, with key parameters presented in Table 1. A lateral and longitudinal coupled controller is designed using model predictive control for trajectory tracking. Additionally, a simulation interface module (Vehicle Interface) is created to receive various inputs, including lateral and longitudinal control commands, gear information, vehicle position data, key parameter configurations, and essential coordinate transformation matrices. This setup allows for the output of the vehicle’s current speed, position, and acceleration, facilitating closed-loop simulation.

**Figure 2:**
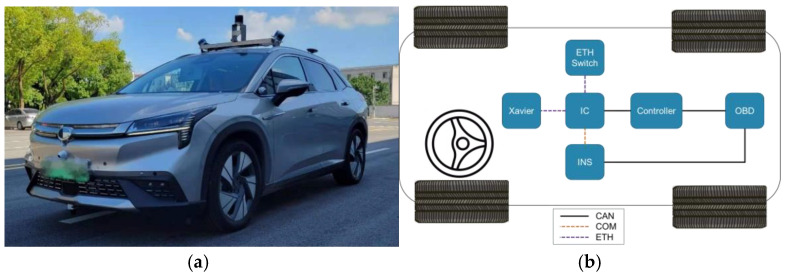
Autonomous driving platform. (**a**) Vehicle test platform. (**b**) Communication topology diagram.

To perform real-vehicle validation, a perception system, localization, mapping, and decision-making control systems are deployed using a specific brand of electric passenger car, as shown in Figure 2a,b, which show the autonomous driving vehicle communication topology diagram, which employs the related hardware component. The improved CILQR method is implemented on an industrial computer (IC), which communicates with the vehicle’s OBD to gather essential vehicle status information and obtains upstream inputs for trajectory planning via Ethernet (ETH). This facilitates trajectory planning, with the deployed trajectory tracking controller calculating the longitudinal and lateral control quantities. The IC transmits these control quantities to the vehicle’s chassis actuators through the On-Board Diagnostics (OBDs) interface for trajectory tracking control, thus completing real-vehicle testing. Xavier is primarily responsible for processing LiDAR data to generate point cloud information for autonomous vehicles. The INS provides precise positioning information for the vehicle.

### 3.1. Simulation Experiment

**Figure 3:**
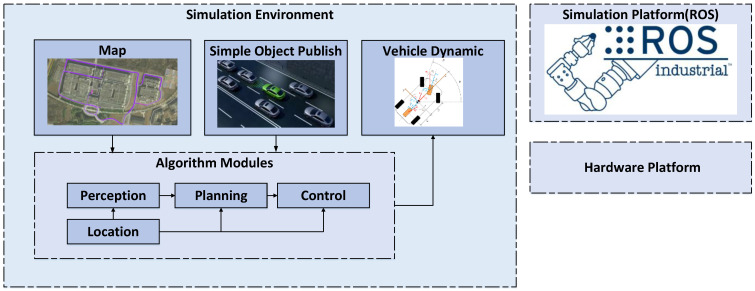
Autonomous driving platform.

A Linux-based operating system with ROS is deployed, enabling the creation of a master node and the definition of interfaces and message structures for key algorithm modules to facilitate seamless inter-node communication. High-precision maps are imported, and obstacle traffic flow information is generated using simple object publish. This setup triggered data streams for the algorithm modules. Control signals are then transmitted to the vehicle dynamics model, facilitating closed-loop simulation testing, as illustrated in Figure 3. Simulation debugging provided the weight parameters listed in Table 2, revealing that $p_{ax_{2}}$, $p_{{\dot{\kappa }}_{2}}$, $p_{{\dot{\kappa }}_{4}}$ have a pronounced impact on driving style. Excessively aggressive or conservative strategies notably affect both traffic efficiency and the sense of safety.

#### 3.1.1. Continuous Nudge Static Obstacle Scenario

**Figure 4:**
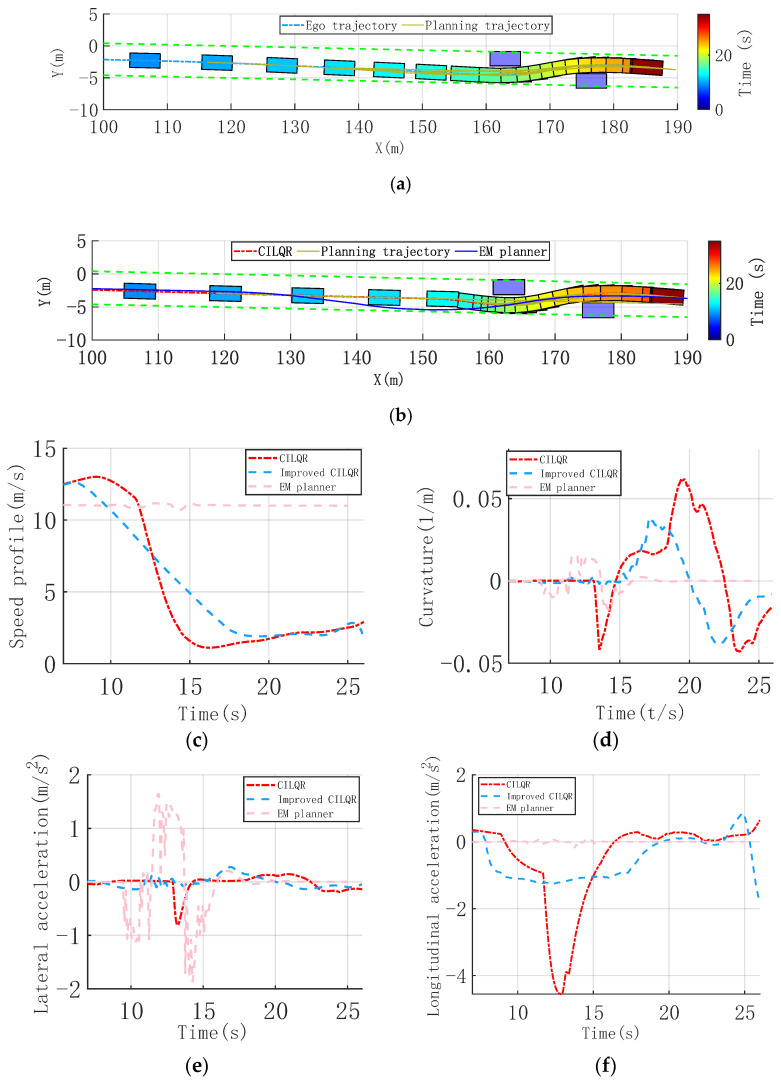
Continuous nudge static obstacle scenario, trajectory, and vehicle dynamic comparison profiles. (**a**) Time sequence diagram of improved CILQR trajectory planning. (**b**) Time sequence diagram of CILQR trajectory planning. (**c**) Speed profile. (**d**) Curvature of Trajectory. (**e**) Lateral acceleration. (**f**) Longitudinal acceleration.

Urban environments, like narrow roads and static obstacles, with limited detour space, are quite familiar. A single-lane scenario is established, with two static vehicles acting as obstacles on either side of the lane. The ego vehicle travels at a high speed, and a detour trajectory on the narrow road is required, necessitating the planning of a reasonable and continuous obstacle-avoidance trajectory for a smooth and comfortable trajectory, as shown in Figure 4a,b.

It shows that the initial trajectory optimization using the hybrid A* method enabled the improved CILQR method to plan slight heading angle adjustments as the vehicle approached obstacles. This led to minor lateral adjustments, avoiding large lateral trajectory shifts and related discomfort. Adaptive adjustment of the weight parameters significantly enhanced the longitudinal speed planning. Starting from 8 s, the speed gradually decreased in a linear fashion, producing a smoother and more continuous speed curve. This avoided sharp changes in lateral and longitudinal acceleration and jerk, ensuring comfort throughout the drive. Consequently, the average speed increased, improving passage efficiency. The time to navigate around the last obstacle improved from 21.3 s to 18.9 s, as shown in Figure 4c. Figure 4d indicates that during the obstacle avoidance process, the trajectory heading angle changes with the improved CILQR method are smoother. In the X = [150, 160] range, the lateral maneuver is smaller, with a peak heading angle of 0.056 rad. The planned curvature remains low, not exceeding 0.035 m^−^¹, and it eliminates oscillations in the curvature due to avoidance needs. The maximum lateral and longitudinal accelerations are kept below 0.039 m/s^2^ and 1.2 m/s^2^, respectively, as shown in Figure 4e,f.

To enable comparison with mainstream methods, the EM planner, based on the literature [36], is implemented. It employs dynamic programming for path planning in the Frenet coordinate system and quadratic programming for speed planning, effectively decoupling the spatio-temporal joint planning problem. The EM planner relies on a sampling-based approach for path planning, demonstrating effective obstacle avoidance in the initial simulation phase. However, stability issues inherent to sampling-based methods lead to curvature oscillations. Combined with high vehicle speeds, this causes significant lateral acceleration, adversely affecting comfort. In contrast, the CILQR method incorporates deceleration planning, maintaining comfort even with larger curvature changes. This underscores the drawbacks of hierarchical decoupled trajectory planning in addressing unified spatio-temporal constraints in these scenarios.

#### 3.1.2. Dynamic Cut-In with Narrow Corridor Scenario

**Figure 5:**
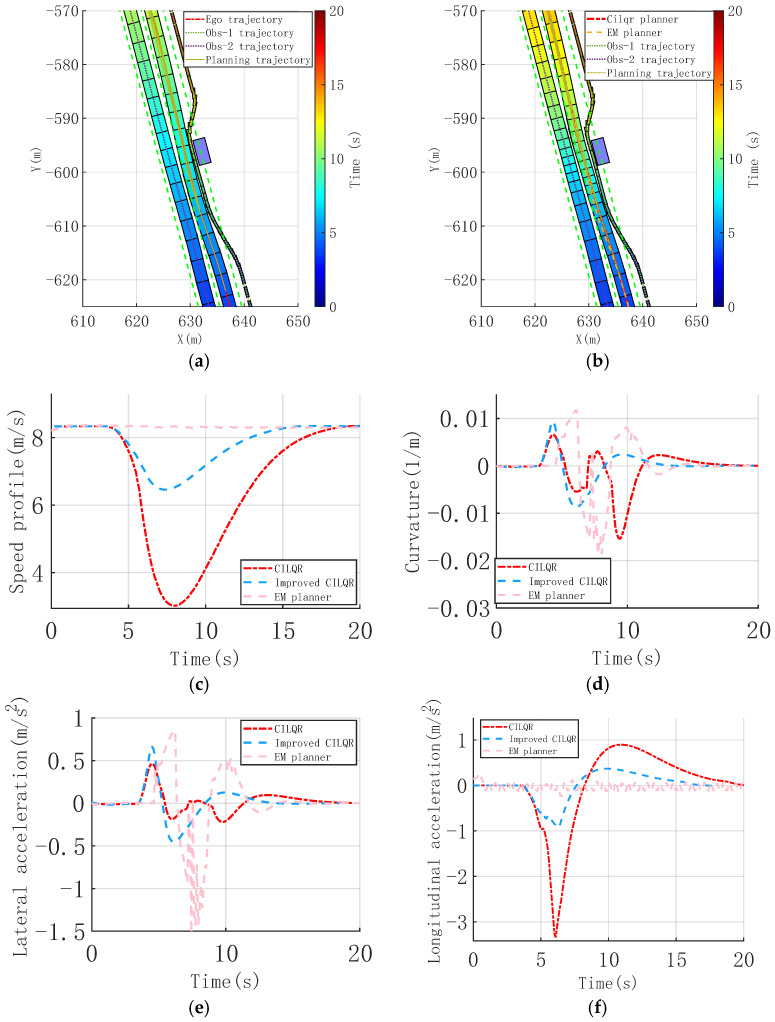
Dynamic cut-in obstacle collision avoidance scenario, trajectory, and vehicle dynamic comparison profiles. (**a**) Time-sequence diagram of improved CILQR trajectory planning. (**b**) Time sequence diagram of CILQR and EM trajectory planning. (**c**) Speed profile. (**d**) Curvature of Trajectory. (**e**) Lateral acceleration. (**f**) Longitudinal acceleration.

Mixed traffic flows, especially with non-motorized vehicles like e-bikes or pedestrians, often present high flexibility and unpredictability. In scenarios with limited space for maneuvering, handling dynamic obstacles that suddenly merge in front of the ego vehicle requires maintaining safety while ensuring efficient passage. A three-lane scenario is created, as shown in Figure 5a,b. It includes two dynamic obstacles: a dynamic vehicle traveling alongside the ego vehicle and an e-bike nudge around a static obstacle. The e-bike’s maneuver and the parallel dynamic vehicle create a merging situation, resulting in a critical space for collision avoidance.

Comparing the ego vehicle’s trajectory time sequence reveals that when the e-bike cut in the ego lane (around 5 s), the improved CILQR method plans the lateral offset trajectory to lead the vehicle to make a slight lateral shift, effectively reducing collision risks. The ego vehicle overtakes the e-bike after 8 s, gradually returning to the lane centerline. The simulation time of the proposed method improved from 20 s to 17.2 s, allowing the vehicle to reach the target point 2.8 s earlier.

Figure 5c,d show that adjusting the adaptive barrier function results in a smoother and more continuous trajectory curvature. The longitudinal speed profile also exhibits significant convergence, decreasing the vehicle’s maximum deceleration from −3.60 m/s^2^ to −0.95 m/s^2^, as depicted in Figure 5f. Although a lateral acceleration of 0.51 m/s^2^ is observed, as shown in Figure 5e, the absolute magnitude is small, and the resulting jerk is minimal, thus having a limited impact on comfort performance.

Similar to the last scenario, the EM planner exhibits substantial curvature oscillations during path planning. This occurs because the longitudinal speed planning does not factor in the comfort costs induced by curvature variations, leading to the maintenance of high vehicle speeds and, consequently, significant lateral acceleration, which cause a critical impact on comfortable performance.

### 3.2. Real-Vehicle Experiment

#### 3.2.1. Dynamic Overtaking of Oncoming Obstacles Scenario

**Figure 6:**
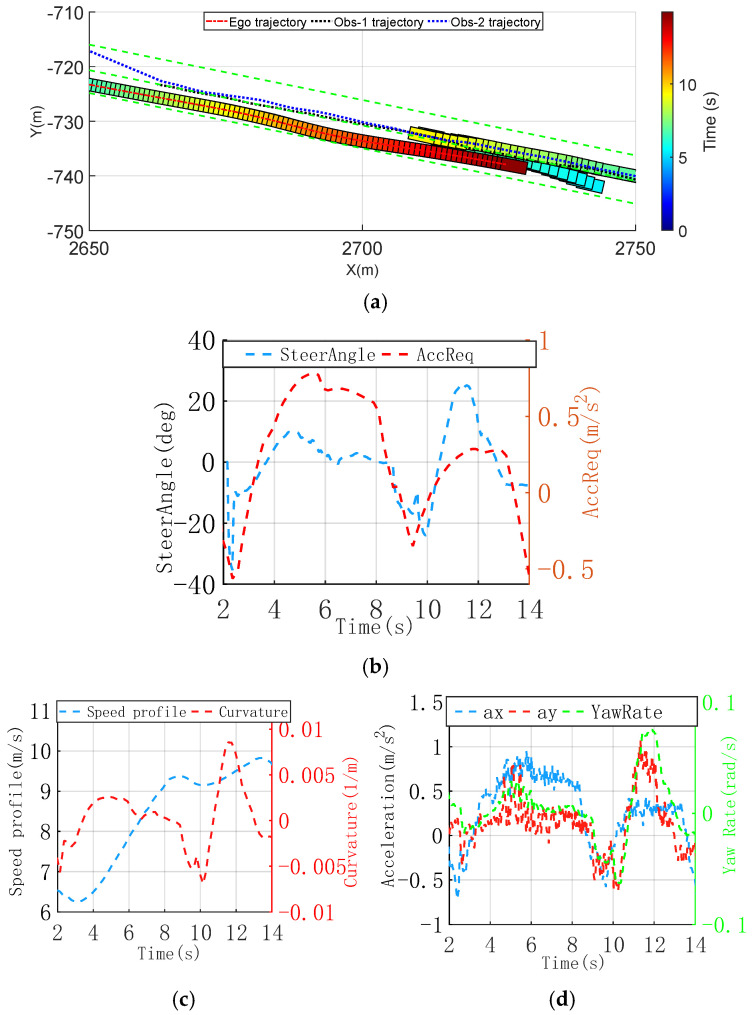
Dynamic overtaking of oncoming obstacles scenario, trajectory, and vehicle dynamic comparison profiles. (**a**) Time-sequence diagram of improved CILQR trajectory planning. (**b**) Vehicle steering wheel angle request and longitudinal acceleration request. (**c**) Speed profile and curvature of trajectory. (**d**) Longitudinal and lateral acceleration of trajectory.

Figure 6a shows two dynamic obstacles moving toward the ego vehicle in the same lane, with one oncoming obstacle straddling the lane and partially encroaching into the ego vehicle’s path, creating a collision risk. To avoid a collision, the ego vehicle plans a lateral shift away from the lane centerline, steering around the obstacle. After the maneuver, the vehicle returns to the lane centerline. To clearly illustrate the obstacle’s movement, only the first 10 s of its trajectory are shown.

Within the initial 8 s, the ego vehicle maintains a normal path within its lane. Significant changes in the trajectory curvature and speed occur. As shown in Figure 6b, during the avoidance maneuver between 8 s and 12 s, adjustments to the steering angle and deceleration occur almost simultaneously, effectively avoiding a collision with the oncoming obstacle. After 12 s, the ego vehicle passes the last obstacle and returns to the lane centerline. The dynamic adjustment of weight parameters allows adaptive tuning of the deceleration weight when the obstacle is near, ensuring a sense of safety during driving. Figure 6c shows the speed profile and curvature changes of the planned trajectory. It indicates that the lateral avoidance maneuver for the obstacle is initiated around the same time. From 9 s, the curvature of the planned trajectory significantly changes, reaching a peak of 9.2 × 10^−3^ m^−1^. Figure 6d shows the vehicle’s dynamic state, with maximum lateral and longitudinal accelerations of 1.05 m/s^2^ and 0.94 m/s^2^, respectively. During lateral maneuvers—whether during the initial avoidance or while returning to the lane center after passing the obstacle—the lateral jerk remains below 2.0 m/s^3^. These dynamic changes ensure a comfortable ride, resulting in more human-like behavior.

#### 3.2.2. Collision Avoidance with Dynamic and Static Obstacles Scenario

**Figure 7:**
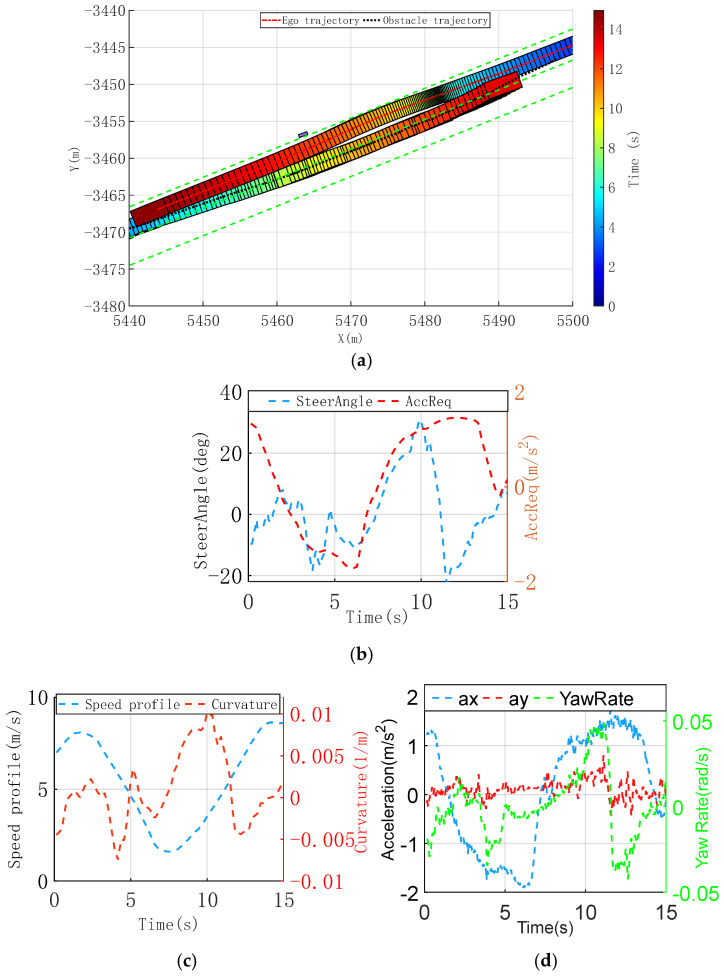
Collision avoidance with dynamic and static obstacles scenario, trajectory, and vehicle dynamic comparison profiles. (**a**) Time-sequence diagram of improved CILQR trajectory planning. (**b**) Vehicle steering wheel angle request and longitudinal acceleration request. (**c**) Longitudinal and lateral acceleration of trajectory. (**d**) Longitudinal and lateral acceleration of trajectory.

Figure 7a shows a scenario involving both dynamic and static obstacles. The ego vehicle confronts an opposite dynamic obstacle in the same lane, while a static obstacle, a pedestrian, partially blocks the ego vehicle’s path. The planned trajectory should meet safety requirements while maximizing comfort and enhancing traffic efficiency. Consequently, the ego vehicle must assess the available space for maneuvering. If the space is inadequate, it must decelerate and brake until sufficient room is available to execute lateral avoidance maneuvers, thus enhancing the safety of other road users and passengers.

Figure 7b shows that the ego vehicle decelerates to reduce collision risk with the static obstacle before 7.2 s, adjusting its trajectory in the ego right direction to increase lateral space for avoiding the oncoming dynamic obstacle. This adjustment results in a lower speed profile, as shown in Figure 7c. Ego vehicle crossing with the oncoming vehicle, with the longitudinal acceleration request according to the speed profile, and simultaneous nudge around the static obstacle.

Figure 7d shows the vehicle’s dynamic states. Although the ego vehicle experiences a peak longitudinal deceleration of nearly −1.8 m/s^2^, the extended deceleration period leads to a gentle longitudinal jerk of −3.3 m/s^3^, maintaining driver comfort. During the dynamic obstacle avoidance, the limited lateral space results in moderate lateral acceleration with minimal impact on comfort. Even in the acceleration phase, the lateral acceleration remains below −0.8 m/s^2^, and the lateral jerk does not exceed 2.01 m/s^3^.

### 3.3. Analysis of Results

Human-like behavior is a key characteristic for enhancing autonomous driving performance. Scholars have investigated metrics for human-like behavior in autonomous driving, generally considering two dimensions: vehicle dynamics and decision-making in relation to obstacles. Given that trajectory planning mainly influences the vehicle’s longitudinal and lateral motion characteristics, it is important to define human-like metrics based on the vehicle’s dynamic features.

Drawing on Makoto Yamakado’s research on the operational behaviors of professional drivers [37], it is evident that professional driving incorporates more simultaneous longitudinal and lateral actions, leading to smoother longitudinal and lateral accelerations. The G-G plot of professional drivers typically resembles a circular shape, suggesting increased coupling between longitudinal and lateral operations. Consequently, the following human-like metrics should be established:

(26) $$f_{hl}=\sum_{i=0}^{N}\frac{\left|q_{a_{x}}\left|a_{x}\right|-q_{a_{y}}\left|a_{y}\right|\right|}{\sqrt{a_{x}^{2}+a_{y}^{2}}},a_{x}<a_{x\_max},a_{y}<a_{y\_max}$$

where $q_{a_{x}},q_{a_{y}}$ denote the calibration parameter for tuning the driving style, like aggressive or moderate. The equation indicates that a smaller $f_{hl}$ value signifies a reduced error in longitudinal and lateral acceleration. This means that the operational point is closer to the diagonal of the G-G diagram, leading to a trajectory profile that appears more circular, which means the human-like character is more obvious. Furthermore, a quantitative analysis of comfort must consider trajectory planning metrics, such as curvature and curvature rate, as well as vehicle dynamic states, including lateral and longitudinal acceleration and yaw rate.

As shown in Table 3, analysis of simulation experiments indicates that the improved CILQR method results in significantly smoother trajectories across two simulation scenarios. The maximum curvature and rate of change in curvature decreased by 38.37% and 67.71% on average, while the maximum longitudinal deceleration dropped by 66.78%. Although lateral acceleration and yaw rate increased slightly under certain conditions, lateral comfort remained consistent. Human-like driving indicator analysis indicates improvements of 9.52% and 23.17% across two simulation conditions. Additionally, traffic efficiency increased by 11.3% and 14.0% in the respective scenarios.

Real-vehicle experiments analysis yield similar conclusions. The planned trajectory curvature and curvature change rates in both test scenarios remained low, staying below 1.0 × 10^−2^ m^−^¹ and 4.7 × 10^−3^ m^−2^. This balance ensures high traffic efficiency while maintaining good lateral and longitudinal comfort performance. In interactive scenarios, human-like driving indicators remained below 0.70, providing an enhanced human-like driving experience. The improved CILQR method’s 99% iteration number $N_{99th}$ decrease almost 45.6%, hence computation time $T_{99th}$ achieved an average speedup of 39.29%, meeting real-time trajectory planning requirements. Notably, each trajectory contains 100 waypoints and extends no more than 200 m.

## 4. Conclusions

This study proposes an improved CILQR method featuring three key features to optimize spatio-temporal joint trajectory planning in complex urban scenarios. The barrier function is optimized, a dynamic weight adjustment strategy is introduced, and the initial trajectory is refined using the hybrid A* method. The trajectory planning problem is formulated using a cost function and constraints, and an autonomous driving simulation and vehicle platform are developed. A human-like indicator is defined to evaluate the effectiveness of the improved CILQR method in spatio-temporal joint planning for complex urban scenarios. The main conclusions of this study are as follows:

(1) First, by optimizing the barrier function, the CILQR method achieves more efficient constraint handling. This enhancement allows for better trajectory adaptation while maintaining safety and comfort, leading to improved numerical stability and a 12.65% average increase in traffic efficiency in complex scenarios.
(2) Second, the adaptive weight adjustment strategy enhances the CILQR method’s adaptability across different scenarios, improving lateral and longitudinal control coordination and increasing the human-like driving indicator by 16.35%.
(3) Integrating the hybrid A* method for initial trajectory preprocessing improves solution efficiency, reducing computation time by 39.29%.

Future study will focus on refining evaluation methods for human-like metrics, providing guidance for further optimization. Additionally, we plan to develop human-like models with diverse driving styles using machine learning, enabling adaptive weight adjustments to customize trajectory planning for individual driver preferences.

## Figures and Tables

**Table 1:** Vehicle key parameters.

Parameter	Description	Value	Unit
m	Vehicle mass	2063.4	kg
$l_{f}$	Front axle distance	1351.8	mm
$l_{r}$	Rear axle distance	1548.2	mm
$C_{f}$	Equivalent front axle cornering stiffness	100,640	N/rad
$C_{r}$	Equivalent rear axle cornering stiffness	100,640	N/rad
$R_{tire}$	Equivalent tire rolling radius	366	mm
$I_{z}$	Yaw moment of inertia about the Z-axis	3347.8	$kg\cdot m^{2}$
$V_{a}$	Vehicle length	4766	mm
$V_{b}$	Vehicle width	2267	mm
$V_{c}$	Vehicle height	1684	mm
$G_{x}$	Longitudinal distance from the center of mass to the rear axle	1549	mm
$G_{y}$	Lateral position of the center of mass	5	mm
$G_{z}$	Vertical position of the center of mass	596	mm
$f_{edge}$	Distance from the front bumper to the rear axle center	3829	mm
$r_{edge}$	Distance from the rear bumper to the rear axle center	937	mm
$S_{ratio}$	Steering ratio between front wheels and steering wheel	16.7	/

**Table 2:** Calibratable weights and matrices.

Adaptive Weighting Parameters	Value	Cost Function Weighting	Value
$p_{ax_{1}}$	1.2	$w_{r}$	2.5
$p_{ax_{2}}$	0.1	$w_{vy}$	12
$p_{ax_{3}}$	20	$w_{ay}$	18
$p_{{\dot{\kappa }}_{1}}$	120	$w_{jy}$	5
$p_{{\dot{\kappa }}_{2}}$	0.12	$w_{vx}$	25
$p_{{\dot{\kappa }}_{3}}$	600	$w_{jx}$	3
$p_{{\dot{\kappa }}_{4}}$	0.05		
$p_{{\dot{\kappa }}_{5}}$	300		

**Table 3:** The comfort and human-like indicators for various methods in both real vehicle and simulation scenarios, along with the 99th% computation time.

Scenarios		Comfortable Indicator					Human-Like Indicator	Computation Cost	
		$\kappa \;[m^{-1}]$	$\dot{\kappa }\;[m^{-2}]$	$a_{x}\;[m/s^{2}]$	$a_{y}\;[m/s^{2}]$	ω [deg/s]	$f_{hl}$	$T_{99th}\;[ms]$	$N_{99th}$
Simulation 1	CILQR	6.2 × 10^−2^	4.8 × 10^−2^	−4.55	−0.81	−0.14	0.63	48.34	55
	Improved CILQR	−3.8 × 10^−2^	2.3 × 10^−2^	−1.77	0.28	0.09	0.57	24.10	25
Simulation 2	CILQR	−1.5 × 10^−2^	−1.2 × 10^−2^	−3.34	0.47	−0.06	0.82	44.71	49
	Improved CILQR	9.4 × 10^−3^	−2.0 × 10^−3^	−0.92	0.67	0.08	0.63	29.34	29
Vehicle Test 1		8.5 × 10^−3^	−3.3 × 10^−3^	−1.31	1.27	0.22	0.51	30.15	28
Vehicle Test 2		1.0 × 10^−2^	4.7 × 10^−3^	−1.91	0.80	0.05	0.68	33.88	31

Both simulation and real vehicle tests used the same software system and hardware platform.

## Data Availability

The data that has been used in this study is confidential.
